# Errors in Dr. Gay’s Hospital Notes for Hydrocele.in Our Last Number

**Published:** 1871-07

**Authors:** 


### Errors in Dr. Gay's Hospital Notes for Hydrocele. in our last number

Our printer and proof reader made Dr. Gay operate upon a *female* instead of a *private* patient, and on page 413, 2d line from top, there should have been a dash between iodine and fluid. This much we take great pleasure in correcting, as arising from our own neglect. We suppose also, that his cases and remarks are open for review. First case operated upon by seton required second operation by tapping to relieve tension. Second case kept in seton thirty days, parts became much enlarged, abscess formed, and patient left hospital before testicle resumed normal shape. Third case was a private patient who had considerable pain and inflammation, but had complete cure. Fourth case, also a private patient, referred to in his remarks, "terminated favorably." These cases were operated upon by the introduction of a seton, the usual manner of doing which, he describes. Upon such data he makes the following

"REMARKS.—Of the several operations for the radical cure of this affection, the one here employed and described, perhaps, presents as many advantages and as few objections as any other. It is certainly as safe and as easy of execution as any, and I know of no reason why it is not as efficient as any. It is attended with as little pain, both during and after the operation, as any of the various methods now employed. If sufficient number of cases should prove the operation inefficient, then there might be added to it or employed along with it, the injection of iodine. Before the cannula is removed a drachm of this tincture might be thrown into the sac through the cannula. If any advantage could possibly result in this double method, it would arise from the fact of the more speedy excitation of the inflammatory process. I think the better way of operating by this method is to insert the seton first and immediately thereafter draw off the water with the trocar, but the fluid would escape even without the employment of the trocar, it will dribble away, and in time escape, but the objection arises, to the non-employment of the trocar, which will be at once anticipated, viz., the delay of the inflammatory process.

Two or three days is, doubtless, sufficiet length of time for the seton to remain, since inflammation will have attained in degree and amount to effect the closure of the sac.

Whether this method of operating for hydrocele is to take precedence over that of swabbing out the sac with iodine, or the other operation of laying open the sac entirely and packing it with lint, thus allowing the healing process to commence from below and within, remains an open and moot question. I have reported the cases in the order in which they occurred with a view to assist in solving the question, and if possible to substitute, for an operation somewhat dangerous, quite painful and protracted in its healing processes, one comparatively painless, simple of execution, little dangerous, and with duration of after treatment much abridged."

These remarks would almost lead us to infer that he is proposing a new operation, the advantages of which have not yet been fully tested, or is endeavoring to establish superior advantages in a plan of operation which had not before been much practiced. We perhaps infer this partly because he makes no reference to the general fact that this operation was anciently, almost wholly employed by surgeons, and as universally abandoned for the more recent plan by injection, as introduced by Sir J. R. Martin of Calcutta, or perhaps by Sir James Earl, about 1791. The oparation by seton originated with the Arabians, and was much in vogue in the fourteenth century, according to Dr. Gross. The progress of his own cases so far as they show anything, would seem to favor very greatly the operation by injection, if, according to statistics, only from one to three per cent fail by it, of radical cure.

The question of treatment of hydrocele, is presented so perfectly in accordance with our own views and teachings by Erichsen, in his recent large work on surgery, that we quote the paragraph on that subject, and leave our readers to their own conclusions, guided as most of them are, by ample personal experience, as to what is really the best plan of procedure for the radical cure of hydrocele :—

"The *curative treatment* has for its object the excitation of a sufficient degree of inflammation in the tunica vaginalis to restore the lost balance between secretion and absorption; but it is not necessary that the serous cavity should be obliterated by adhesions between its opposite sides, though these not unfrequently take place. The means by which the surgeon sets up this inflammation are either the introduction of a small seton into the tunica vaginalis, or throwing a stimulating injection into that cavity after tapping it. Whichever plan is adopted, a certain amount of inflammation ought to be set up. This is always attended by considerable swelling of the testis, and by the effusion of a fresh quantity of fluid into the tunica vaginalis. As this is absorbed, the part gradually resumes its normal bulk, and the disease will probably not return.

"In order that the radical cure, in whichever way undertaken, should be safe and efficient, it is necessary, in the first instance, that the disease should have been allowed to attain a chronic condition, more particularly if the hydrocele have been of rapid growth. In order to prevent its attaining too large a size, it will be well to adopt palliative tapping once or twice before attempting the radical cure. Care must also be taken to remove all inflammation and tenderness about the testis, before having recourse to this means of treatment. If attention be not paid to this, recurrence of the hydrocele will probably ensne. After the proper amount of inflammation has been set up, it will be well to treat the patient as if he were suffering under an ordinary attack of orchitis, confining him to the bed or to the couch for a few days; indeed, care in the after-treatment is of very considerable importance in securing a favorable result.

"The treatment by *injection* is that which is commonly employed. It consists in tapping the tumor in the usual way, and then throwing a sufficient quantity of stimulating fluid into the tunica vaginalis through the canula, so as to excite a proper amount of inflammation in it. The liquids that are employed are generally either port wine, or a solution of the sulphate of zinc of the strength of ʒj to ℥xij, or most commonly the tincture of iodine. If the port wine or a solution of the sulphate of zinc be employed, a sufficient quantity partly to distend the sac should be injected from an India-rubber bottle or brass syringe that can be adapted to the canula; six or eight ounces are commonly required for this purpose, and it should be allowed to remain in some minutes before being evacuated.

*Injection of Iodine.*—The injection of tincture of iodine, originally introduced by Sir J. R. Martin, whilst practising at Calcutta, is now commonly preferred as a more certain and a safer mode of treatment than any other. It is usually sufficient to inject about one or two drachms of the pure tincture. It should be left in for a few minutes, in proportion to the amount of pain it occasions, and then allowed to escape. (I believe the operation to be more likely to succeed if the tincture of iodine be allowed to remain in the sac as recommended by Prof. Syme. Tne amount used should vary from one to three finidrachms according to the size of the hydrocele.—A.) The canula used for this purpose should be made of platinum and not of silver, which is apt to become corroded and made brittle by the action of the Iodine. A good deal of inflammation will usually be set up, on the subsidence of which, the cure will be found to have been effected.

"Useful as the iodine injection is, it sometimes fails in producing a radical cure of hydrocele. This is attributable to two causes: the first is, that in some cases sufficient inflammation is not set up to induce that condition of the tunica vaginalis which is necessary for a radical cure. It is well known that when a hydrocele is radically cured by injection, it is so, not by any adhesion, taking place between the two opposite snrfaces of the tunica vaginalis and a consequent obliteration of its cavity, but by the inflammation that is artificially induced exciting such a modification to this membrane as to restore the balance between the secretion and absorption of the fluid by which it is naturally lubricated. Now, in some cases, sufficient inflammation is not induced by the introduction of the irritating fluid to restore the natural balance between these two fuuctions of the membrane; and the tunica vaginalis gradually fills again after the injection, as it would after the simple operation of tapping. It occasionally happens that the patient may suffer excruciating agony at the time of the injection, from the contact of the stimulating fluid with the surface of the testis, and yet little or no inflammation may be excited. The amount of suffering, therefore, at the time of the operation is by no means proportionate to the amount of consecutive inflammation likely to be set up. Indeed. the reverse would appear to be the case in many instances; and I have often observed that, in those cases which progress most steadily to a radical cure, there is but a moderate amount of pain experienced at the time of the injection.

"There is a second way in which injections would appear to fail; a considerable amount of inflammation is excited, and effusion takes place into the tunica vaginalis, which, in the course of three or four days, becomes distended to the same size, or nearly so, that it had obtained previously to the operation; but this effused fluid, instead of being absorbed by the end of the second or third week, remains unchanged in bulk, or absorption goes on to a certain point, and then seems to be arrested; the tunica vaginalis remaining distended with a certain quantity of fluid.

"The proportion of cases in which the iodine injection fails to bring about a radical cure of the hydrocele is variously estimated by different surgeons. Thus Sir J. R. Martin states that in India the failures scarcely amount to 1 per cent.; Velpeau calculates them at 3 per cent. I am not aware that any statistics of this mode of treatment in this country have been collected; but the general opinion of surgeons would appear to be decidedly in its favor, as being the most successful as well as the safest plan of treatment that has yet been introduced. In this opinion I fully coincide; yet I think it by no means improbable that the success of the iodine injection in this country might not prove to be quite so great as is generally believed. I have, during the last few years, seen a considerable number of cases of simple hydrocele of the tunica vaginalis, both in hospital and in private practice, in which a radical cure had not been affected, although recourse had been had to the iodine injection by some of the most careful and skilful surgeons of the day, as well as by myself.

"One circumstance connected with the injection of tincture of iodine into the tunica vaginalis deserves note. It is that although in some cases it occasions but little pain, in other instances the suffering induced by it is of the most severe and agonizing character—more so than follows the introduction of any other of the ordinary stimulants into the tunica vaginalis.

"*Seton.*—The cure by the introduction of a seton, though formerly much employed, is seldom practised at the present day, chiefly on account of the danger of exciting too much inflammation. It may, however, conveniently be employed in the true hydroceles of children, and in some of those cases in which the injection fails, if practised in the manner that will be immediately described. There can be no doubt that, as a first remrdy, iodine injection is preferable to the seton, in the treatment of hydrocele; but when the injection has failed, and this from no want of care on the part of the surgeon, or of attention to the after-treatment of the case, but apparently from insufficient inflammatory action having been set up in the tunica vaginalis to restore the lost balance between secretion and absorption in this membrane, the seton will, I think, be found to be the most certain means of accomplishing our object. It is true that several objections may be urged to the use of the seton; it requires much watching and care, and is occasionally apt to excite a dangerous amount of inflammation in the areolar tissue of the scrotum; and these objections are, to my mind, sufficient valid to prevent our employing it as the ordinary treatment for the radical cure of hydrocele. But it must be remembered, that the particular cases to which I am now alluding are those in which ordinary means have proved insufficient to excite proper action, and in which, consequently, it would appear as if a greater amount of irritation could safely be borne. Indeed, nothing is more remarkable than the difference in the intensity of the inflammation that is set up in different individuals by the means that are commonly employed in the treatment of hydrocele. In some cases the most irritating injections may be thrown into the tunica vaginalis, or a seton may be drawn through the scrotum and left there for days, not only without giving rise to any injurious inflammation, but without setting up sufficient action to bring about a cure of the disease; whilst in other instances simple tapping may effect a radical cure, or may give rise to such an amount of irritation as to terminate in a fatal sloughing of the scrotum.

"The seton that I employ in these cases is composed of one or two threads of dentist's silk. It may be introduced by means of a nævus-needle, the fluid of the hydrocele being allowed to drain away through the punctures thus made; or, far better, by tapping the hydrocele, and then passing a needle about six inches long, armed with the seton, up the canula, drawing it through the upper part of the scrotum, and then removing the canula, cutting off the needle, and knotting the thread loosely. [The seton may be conveniently introduced by replacing the trocar and making a second puncture, this time from within outwards. An eyed probe carrying the thread may then be passed throug the canula and upon the withdrawal of the latter the seton will be in place.—A.] The thread should not be removed until the scrotum swells and becomes red, with some tenderness of the testis and effusion into the tunica vaginalis. When these effects have been produced, it may be cut and withdrawn, and the case treated in the same way as when the radical cure has been attempted by iodine injection; viz., by rest and antiphlogistic treatment. The length of time during which the seton must be left in before sufficient, or even any inflammatory action is produced, varies very considerably. In most instances, the proper amount of inflammation is excited in from twenty-four to thirty hours; but in other cases the seton may be left in for ten or twelve days, giving rise to but little inflammation although a radical cure may result."

